# Corticotropin-Releasing Hormone Receptor 2 Gene Variants in Irritable Bowel Syndrome

**DOI:** 10.1371/journal.pone.0147817

**Published:** 2016-01-25

**Authors:** Hazuki Komuro, Naoko Sato, Ayaka Sasaki, Naoki Suzuki, Michiko Kano, Yukari Tanaka, Yumi Yamaguchi-Kabata, Motoyori Kanazawa, Hitoshi Warita, Masashi Aoki, Shin Fukudo

**Affiliations:** 1 Department of Behavioral Medicine, Tohoku University Graduate School of Medicine, Sendai, Japan; 2 Department of Neurology, Tohoku University Graduate School of Medicine, Sendai, Japan; 3 Frontier Research Institute for Interdisciplinary Sciences, Tohoku University, Sendai, Japan; 4 Department of Integrative Genomics, Tohoku Medical Megabank Organization, Tohoku University, Sendai, Japan; 5 Graduate School of Medicine, Tohoku University, Sendai, Japan; 6 Department of Psychosomatic Medicine, Tohoku University Hospital, Sendai, Japan; Medical University of Gdańsk, POLAND

## Abstract

**Background:**

Corticotropin-releasing hormone (CRH) plays an important role in the pathophysiology of irritable bowel syndrome (IBS) and regulates the stress response through two CRH receptors (R1 and R2). Previously, we reported that a *CRHR1* gene polymorphism (*rs110402*, *rs242924*, and *rs7209436*) and haplotypes were associated with IBS. However, the association between the *CRHR2* gene and IBS was not investigated. We tested the hypothesis that genetic polymorphisms and haplotypes of *CRHR2* are associated with IBS pathophysiology and negative emotion in IBS patients.

**Methods:**

A total of 142 IBS patients and 142 healthy controls participated in this study. Seven single nucleotide polymorphisms (SNPs) of the *CRHR2* gene (r*s4722999*, *rs3779250*, *rs2240403*, *rs2267710*, *rs2190242*, *rs2284217*, and *rs2284220*) were genotyped. Subjects' psychological states were evaluated using the Perceived-Stress Scale, the State-Trait Anxiety Inventory, and the Self-Rating Depression Scale.

**Results:**

We found that *rs4722999* and *rs3779250*, located in intronic region, were associated with IBS in terms of genotype frequency (*rs4722999*: *P* = 0.037; *rs3779250*: *P* = 0.017) and that the distribution of the major allele was significantly different between patients and controls. There was a significant group effect (controls vs. IBS), and a *CRHR2* genotype effect was observed for three psychological scores, but the interaction was not significant. We found a haplotype of four SNPs (*rs4722999*, *rs3779250*, *rs2240403*, and *rs2267710*) and two SNPs (*rs2284217* and *rs2284220*) in strong linkage disequilibrium (D′ > 0.90). We also found that haplotypes of the *CRHR2* gene were significantly different between IBS patients and controls and that they were associated with negative emotion.

**Conclusion:**

Our findings support the hypothesis that genetic polymorphisms and haplotypes of *CRHR2* are related to IBS. In addition, we found associations between *CRHR2* genotypes and haplotypes and negative emotion in IBS patients and controls. Further studies on IBS and the CRH system are warranted.

## Introduction

The stress response is an important mechanism that is indispensable for the maintenance of life. Corticotropin-releasing hormone (CRH) is released from the paraventricular nucleus of the hypothalamus under stress and it stimulates the secretion of adrenocorticotropic hormone (ACTH) from the pituitary gland. ACTH acts on the adrenal cortex and promotes the synthesis of glucocorticoid. This system is called the hypothalamic-pituitary-adrenal (HPA) axis [1]. In the hippocampus, hypothalamus, and pituitary gland, there are two receptors that interact with cortisol: the glucocorticoid receptor and mineralocorticoid receptor. When cortisol secretion is increased, the composition and secretion of CRH and ACTH is controlled through these receptors, which results in the suppression of cortisol secretion (negative feedback) [2]. Dysfunction of the HPA axis causes hippocampal atrophy [3] and it is also related to stress-related diseases such as depression, panic disorder, and post-traumatic stress disorder (PTSD) [4–7].

CRH is a polypeptide consisting of 41 amino acid residues [8]. The CRH family consists of several neuropeptides: CRH, urocortin 1, urocortin 2, and urocortin 3. CRH is primarily responsible for regulating and/or initiating stress responses via activation of the HPA axis [9]. Conversely, urocortins play an indispensable role in the recovery response to stress [9]. CRH released in the brain activates the sympathetic system and stimulates the cardiovascular system [10]. In addition, it stimulates sacral parasympathetic outflow and colonic motility [11]. The dysregulation of CRH increases anxiety and depression [12].

There are two G protein-coupled receptors for CRH: CRH receptor 1 (CRHR1, CRF_1_ in IUPHAR nomenclature) and CRH receptor 2 (CRHR2, CRF_2_ in IUPHAR nomenclature). CRHR1 is found at a high density in the brain and pituitary gland, while it is found at a low density in the adrenal gland, ovary, and testis [13–16]. Other than its expression in the brain, CRHR2 is distributed widely throughout peripheral organs such as the heart, skeletal muscle, intestines, and airway epithelium [17–20]. CRH activates both receptors but is more potent at CRHR1. Urocortin 1 can bind both receptors with equal affinity that is 10-fold greater than CRH binding affinity for CRHR1. urocortin and urocortin don’t bind to CRHR1, but CRH, urocortin 2 and urocortin 3 all bind to CRHR2 with equal affinity [21]. The distribution of these receptors and their binding affinities to endogenous ligands in individuals may reflect individual differences in physiological function.

Irritable bowel syndrome (IBS) is a prototypic functional gastrointestinal disorder [22] accompanied by visceral hypersensitivity [23], increased gut reactivity [24], and altered central processing [25] in response to various stressors [26]. A previous study demonstrated that mutual and reciprocal interactions between the brain and gut play a major role in the pathophysiology of IBS [27]. In IBS patients, the exogenous administration of CRH induced robust colonic motility [24]. Electrical stimulation of the rectum induced an increase in motility indices in IBS patients and this response was inhibited by the administration of a CRH antagonist [28]. Therefore, dysfunction of CRH signaling is considered to be related to the pathophysiology of IBS.

CRH plays a stimulatory role in the stress response through the activation of CRHR1, while the specific actions of urocortin 2 and urocortin 3 on CRHR2 may be important for dampening the stress response [4]. In the brain, CRHR1 stimulation causes anxiety, but CRHR2 stimulation induces anxiolysis. In regard to gut motility, CRHR1 stimulation evokes colonic motility, but CRHR2 stimulation inhibits gastric emptying [12]. From these studies, one of the important determinants of the brain-gut interaction in the stress response of IBS patients is signaling via CRHR1 and CRHR2.

The gene encoding *CRHR1* is located on chromosome 17q21.31 and has a length of 51.55 Kb and contains 14 exons. The gene encoding *CRHR2* is located on chromosome 7p14.3 and has a length 48.19Kb and contains 16 exons. There are many reports on the association between gene variants of CRH receptors and stress-related diseases. In *CRHR1*, variation of the gene has been found to be a risk factor for depression after childhood maltreatment [29–33]. A polymorphism of *CRHR1* was reported to be related to recurrent major depressive disorder (MDD) [34]. In regard to *CRHR2*, gene variation may affect the risk of PTSD in women by attenuating the stress response and reducing symptoms of the disorder [35]. A polymorphism in the *CRHR2* gene was also associated with MDD [36]. In a previous study from our laboratory, we showed that genetic polymorphisms and haplotypes of *CRHR1* (*rs110402*, *rs242924*, and *rs7209436*) mediate IBS and related bowel patterns [37]. However, the association between *CRHR2* genotypes and IBS has not been investigated. Therefore, we hypothesized that gene polymorphisms and/or haplotypes of *CRHR2* may be associated with IBS pathophysiology and negative emotion in IBS patients.

## Methods

### Subjects

In total, 142 patients (62 males and 80 females) with IBS who were diagnosed at the Department of Psychosomatic Medicine, Tohoku University Hospital, were enrolled in this study (mean age 22.0 ± 0.2 years; range 18–31). Patients with organic diseases were excluded. In addition, 142 healthy volunteers (74 males and 68 females) were recruited at Tohoku University as controls (mean age 22.0 ± 0.2 years; range 19–31). Subjects without any symptoms or signs during a medical interview and physical examination were identified as healthy controls. There was no significant difference in age, sex ratio among the groups.

IBS patients were diagnosed according to the Rome III criteria [38]. IBS was defined as recurrent abdominal pain or discomfort for at least 3 days per month in the last 3 months associated with two or more of the following symptoms: improvement with defecation, onset associated with a change in the frequency of defecation, and/or onset associated with a change in the form (appearance) of stools. These criteria were fulfilled for the previous 3 months with symptom onset at least 6 months prior to diagnosis. According to the Rome III criteria, IBS was classified as IBS with diarrhea (D), constipation (C), or mixed symptoms of diarrhea and constipation (M). Unclassified IBS patients were classified as IBS-M. All subjects provided written informed consent and this study was approved by the Tohoku University Ethics Committee. Consecutive patients who agreed to participate in this study were enrolled.

Two hundred and twenty-two of 284 participants’ DNA samples were previously reported on polymorphisms of the *CRHR1* polymorphic region [37]. There are apparently different from the targeted genes in this study.

### SNP selection

**Fig 1:**
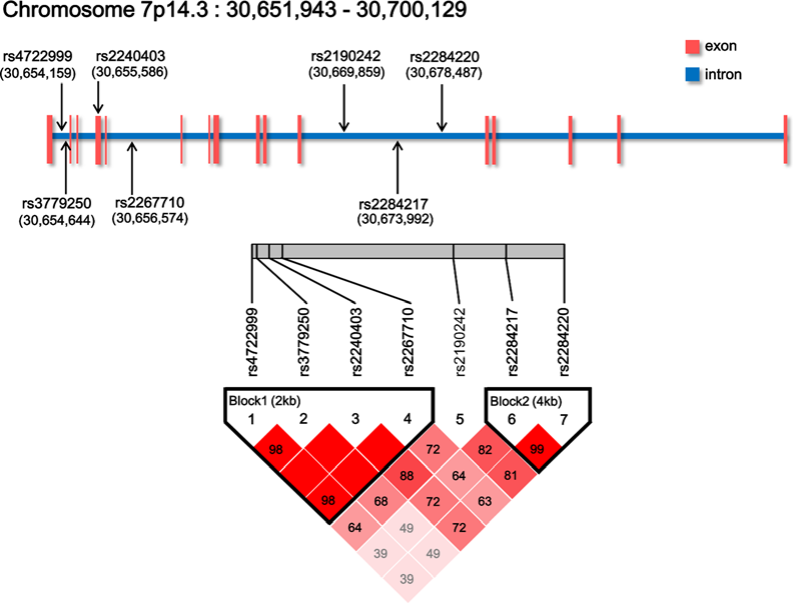
SNPs of the *CRHR2* gene examined in this study. *CRHR2* is located on chromosome 7p14.3. The arrows and the numbers in parentheses indicate the locus of each SNP. *rs2240403* exists in the fourth exon, but six other SNPs exist in intronic regions. These SNPs had a minor allele frequency of >15% in the Japanese population. A linkage disequilibrium (LD) plot was generated using D′. D′ = 1 indicates complete LD. D′ values < 1.0 are shown in the respective squares for the SNP pairs, with darker shades of red representing higher levels of LD.

We used Generic Genome Browser in International HapMap Project (http://hapmap.ncbi.nlm.nih.gov/index.html.en) to search the allele frequency (minor allele frequency > 0.15 in Japanese population). Furthermore, we restricted seven candidate genes, which were selected from single nucleotide polymorphisms (SNPs) for genotyping based on previous literature [35,36,39]. The loci of these SNPs were searched from the dbSNP in the National Center for Biotechnology Information (NCBI, http://www.ncbi.nlm.nih.gov/), and are shown in **Fig 1**.

### Genotyping

Peripheral blood was sampled from the forearm vein of each subject with a heparinized syringe. DNA was then extracted from the lymphocytes using a standard protocol [40]. The concentration of each DNA was diluted to 0.2 μg/μL. Seven SNPs (*rs4722999*, *rs3779250*, *rs2240403*, *rs2267710*, *rs2190242*, *rs2284217*, *and rs2284220*) in the regulatory region of the *CRHR2* gene were genotyped using real-time polymerase chain reaction (PCR).

We use the CFX-96 Touch^TM^ Real-time PCR Detection System (Bio-Rad Laboratories, Inc., Tokyo, Japan). PCR amplification was carried out using probe/primer sets for SNP genotyping that were purchased from Applied Biosystems, Tokyo, Japan (set of C1617682310 for *rs2240403*, C1587286110 for *rs2190242*, C1596058610 for *rs2284217*, C1596058010 for *rs2284220*, C34646010 for *rs4722999*, C2580980110 for *rs3779250*, and C1587287110 for *rs2267710*). PCR amplification was performed using a total volume of 10 μL consisting of 5 μL TaqMan Genotyping Master Mix, 4.4 μL ddH_2_O, 0.5 μL TaqMan SNP Genotyping Assays, and 0.1 μL DNA. After initial denaturation at 95°C for 10 min, amplification was performed for 40 cycles at 95°C for 15 s and 60°C for 1 min. To determine the positive control, sequence analysis of CRHR2 genes was performed using an ABI 3130 Genetic Analyzer (PE Applied Biosystems, Foster City, CA). All procedures were performed according to the manufacturer's instructions.

### Evaluation of psychological state

Participants' psychological states were rated using the Perceived-Stress Scale (PSS; [41], [42]), the State-Trait Anxiety Inventory (STAI; [43], [44]), and the Self-Rating Depression Scale (SDS; [40], [45]). The Japanese versions of STAI, SDS, and PSS have been validated and their reliability has been confirmed [42].

### Statistical analysis

The frequencies of genotypes, alleles, and haplotypes of the *CRHR2* SNPs were compared between the IBS patients and controls, or between patients with different bowel patterns (normal, constipation, mixed, and diarrhea) using the χ^2^-test. The effects of variation in *CRHR2* SNPs and haplotypes on psychological states between the IBS patients and controls were examined with two-way analysis of variance (ANOVA). The effects of variation in *CRHR2* haplotypes on psychological states were examined with one-way ANOVA. Statistical analyses were performed using SPSS Statistics version 21.0 software (IBM, Inc., New York, NY). We used Haploview [46] to determine the LD of the SNPs within the *CRHR2* gene and test for Hardy–Weinberg equilibrium. Results are expressed as the mean ± standard error, and a *P* value < 0.05 was considered to be significant.

## Results

In the 284 samples, genotype distributions were in Hardy–Weinberg equilibrium for all seven SNPs. The genotype frequencies of the SNPs in the IBS group and healthy controls are listed in **Table 1**. The allele frequencies of the SNPs in the IBS group, healthy controls, and the subtypes of IBS patients are listed in **Table 2**.

**Table 1:** Genotypes frequencies for seven *CRHR2* SNPs in the IBS patients and controls.

		Controls n (%) n = 142		IBS Patients n (%) n = 142		*P* value(Control vs. IBS)
		Male (n = 74)	Female (n = 68)	Male (n = 62)	Female (n = 80)	
rs4722999	CC	25 (17.6)	18 (12.7)	15 (10.6)	23 (16.2)	**0.037**
	CT	33 (23.2)	34 (23.9)	39 (27.5)	47 (33.1)	
	TT	16 (11.3)	16 (11.3)	8 (5.6)	10 (7.0)	
rs3779250	CC	27 (19.0)	21 (14.8)	16 (11.3)	28 (19.7)	**0.017**
	CT	32 (22.5)	31 (21.8)	38 (26.8)	44 (31.0)	
	TT	15 (10.6)	16 (11.3)	8 (5.6)	8 (5.6)	
rs2240403	CC	54 (38.0)	37 (26.1)	38 (26.8)	51 (35.9)	0.855
	CT	18 (12.7)	30 (21.1)	24 (16.9)	27 (19.0)	
	TT	2 (1.4)	1 (0.7)	0 (0.0)	2 (1.4)	
rs2267710	CC	28 (19.7)	23 (16.2)	20 (14.1)	32 (22.5)	0.107
	CT	33 (23.2)	32 (22.5)	35 (24.6)	41 (28.9)	
	TT	13 (9.2)	13 (9.2)	7 (4.9)	7 (4.9)	
rs2190242	CC	25 (17.6)	23 (16.2)	21 (14.8)	31 (21.8)	0.518
	AC	38 (26.8)	30 (21.1)	30 (21.1)	41 (28.9)	
	AA	11 (7.7)	15 (10.6)	11 (7.7)	8 (5.6)	
rs2284217	GG	22 (15.5)	22 (15.5)	22 (15.5)	19 (13.4)	0.171
	GA	37 (26.1)	42 (29.6)	24 (16.9)	46 (32.4)	
	AA	15 (10.6)	4 (2.8)	16 (11.3)	15 (10.6)	
rs2284220	AA	21 (14.8)	22 (15.5)	23 (16.2)	19 (13.4)	0.289
	AG	37 (26.1)	41 (28.9)	23 (16.2)	46 (32.4)	
	GG	16 (11.3)	5 (3.5)	16 (11.3)	15 10.6	

*P* value < 0.05 were indicated in bold.

**Table 2:** Allele expression in the controls, IBS patients, and IBS subtypes for seven SNPs of the *CRHR2* gene.

		Controls	IBS Patients				Total
			All	C	M	D	
		n = 142	n = 142	n = 41	n = 36	n = 65	n = 284
rs4722999	C allele -	32	18	9	1	8	50
	C allele +	110	124	32	35	57	234
	T allele -	43	38	7	11	20	81
	T allele +	99	104	34	25	45	203
rs3779250	C allele -	31	16	6	1	9	47
	C allele +	111	126	35	35	56	237
	T allele -	48	44	10	14	20	92
	T allele +	94	98	31	22	45	192
rs2240403	C allele -	3	2	0	2	0	5
	C allele +	139	140	41	34	65	279
	T allele -	91	89	26	23	40	180
	T allele +	51	53	15	13	25	104
rs2267710	C allele -	26	14	5	1	8	40
	C allele +	116	128	36	35	57	244
	T allele -	52	52	12	16	24	104
	T allele +	90	90	29	20	41	180
rs2190242	C allele -	26	19	4	1	14	45
	C allele +	116	123	37	35	51	239
	A allele -	48	52	11	18	23	100
	A allele +	94	90	30	18	42	184
rs2284217	G allele -	19	31	8	9	14	50
	G allele +	123	111	33	27	51	234
	A allele -	44	41	7	10	24	85
	A allele +	98	101	34	26	41	199
rs2284220	A allele -	21	31	8	9	14	52
	A allele +	121	111	33	27	51	132
	G allele -	43	42	7	11	24	85
	G allele +	99	100	34	25	41	199

IBS subtype: C, constipation; M, mixed; D, diarrhea.

**Fig 2:**
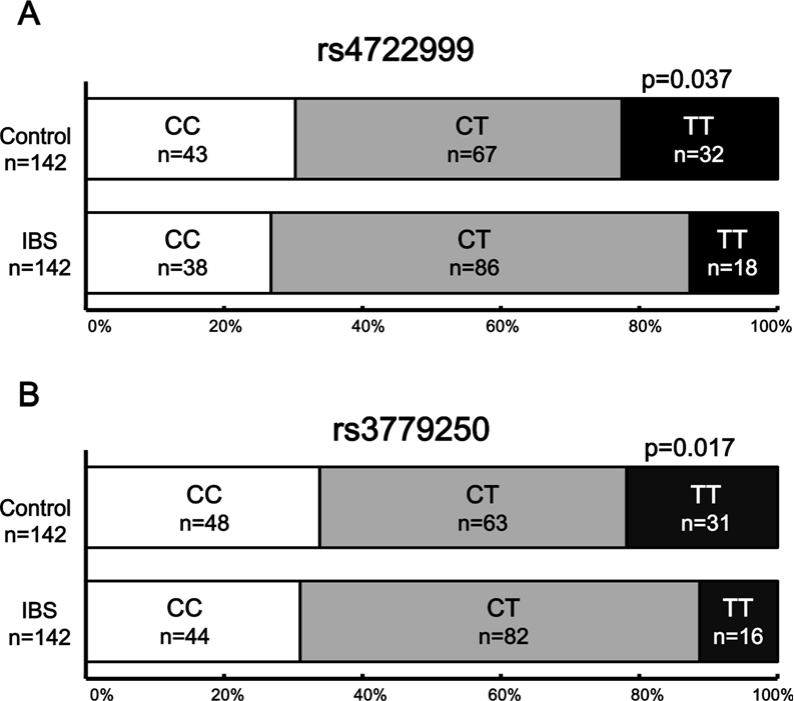
Difference in genotype of *CRHR2* SNPs between the controls and IBS patients. The SNPs *rs4722999* (**A**) and *rs3779250* (**B**) are shown. The SNPs *rs4722999* (*P* = 0.037) and *rs3779250* (*P* = 0.017, χ^2^-test) were significantly different in the IBS patients in comparison with the controls.

We found that *rs4722999* (A) and *rs3779250* (B) were significantly associated with IBS in terms of genotype distribution (*rs4722999*: *P* = 0.037; *rs3779250*: *P* = 0.017) (**Fig 2**). For these two SNPs, there was no significant difference in the frequency of the minor allele, but the frequency of the major allele was significantly different.

**Table 3**shows the scores of the psychological scales in the controls and IBS patients. There was no significant difference in the test scores for the three psychological scales between the IBS patients and controls.

**Table 3:** Perceived stress, depression, and anxiety in the controls and IBS patients.

	Controls		IBS Patients		P value
	n = 142		n = 142		
	Mean	SE	Mean	SE	
PSS	26.7	0.8	28	0.7	0.203
STAI (state)	44.1	0.8	45.2	0.8	0.361
STAI (trait)	47.9	0.9	47.5	0.9	0.739
SDS	39.4	0.7	40.6	0.6	0.23

PSS: Perceived Stress Scale, STAI: State Trait anxiety Inventry, SDS: Self-rating Depression Scale.

**Fig 3:**
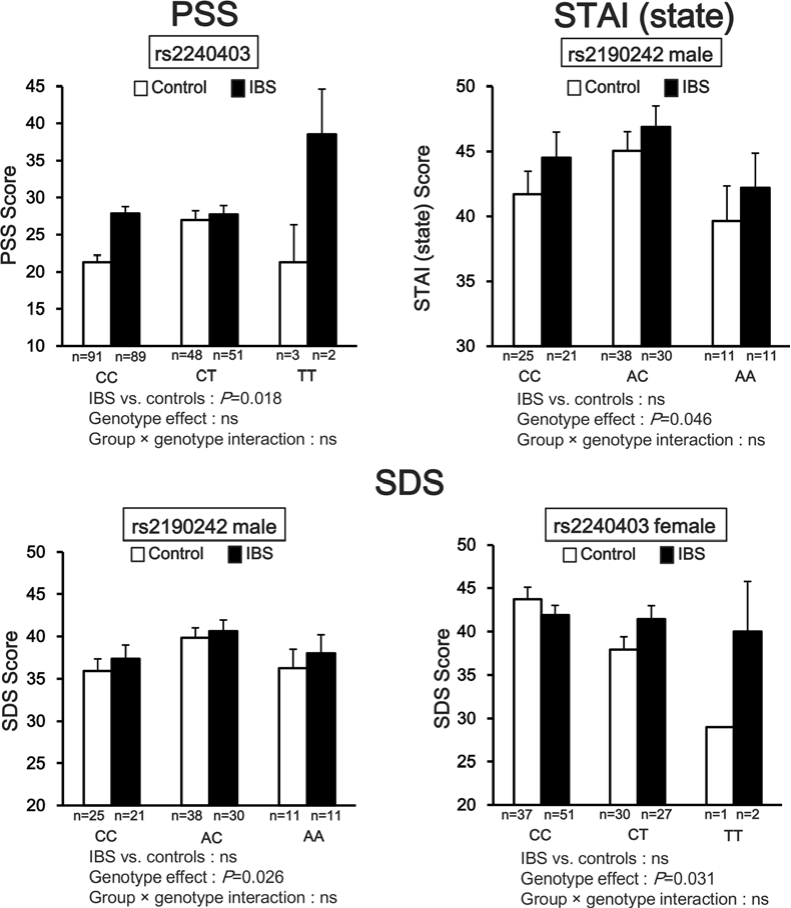
Psychological scales and *CRHR2* SNPs in IBS patients and controls. PSS: two-way ANOVA showed that the IBS patients with *rs2240403* (*P* = 0.018) had significantly higher perceived stress scale scores than the controls. STAI (state): *rs2190242* in males, genotype effect was significant (*P* = 0.046). SDS: *rs2190242* in males and *rs2240403* in females, genotype effect was significant (*rs2190242*: *P* = 0.026; *rs2240403*: *P* = 0.031). In all scale scores, there was no significant group × genotype interaction.

**Fig 3**shows that the three psychological scores were significantly different by *CRHR2* genotype or group (IBS patients vs. controls). According to PSS in *rs2240403*, the IBS patients were significantly higher than the controls (*P* = 0.018), but there was no significant interaction. According to STAI (state) in *rs2190242* in males, there was a significant genotype effect (*P* = 0.046), but there was no significant interaction. According to SDS in *rs2190242* in males and *rs2240403* in females, there was a significant genotype effect (*rs2190242*: *P* = 0.026; *rs2240403*: *P* = 0.031), but there was no significant interaction.

According to the selection criteria, four SNPs (*rs4722999*, *rs3779250*, *rs2240403*, and *rs2267710*) of *CRHR2* with strong D′ (>0.90) were in one linkage block, and two SNPs (*rs2284217* and *rs2284220*) with strong pairwise D′ (>0.90) were in another block (**Fig 1**). The selection criteria for haplotypes used in the haplotype analyses were adjacent SNPs with pairwise D′ > 0.90. Haplotype analysis of the IBS group and healthy controls is shown in **Table 4**. We found that three blocks were positively associated with IBS patients (T-C-C-C: *P* = 0.017; T-C-C-T: *P* = 0.017; C-T-C-C: *P* = 0.043). All haplotypes were found more frequently in the IBS patients than in the controls.

**Table 4:** Haplotype frequencies of the IBS patients and controls in the *CRHR2* gene.

Haplotype	Control—Freq	Case—Freq	*P* value
rs4722999—rs2267710			
TTCT	0.641	0.620	0.806
CCCC	0.754	0.852	0.052
CCTC	0.359	0.373	0.902
TTCC	0.479	0.592	0.074
TCCC	0.479	0.627	**0.017**
CTCT	0.415	0.514	0.122
TTTC	0.155	0.211	0.283
TTTT	0.148	0.190	0.429
CTTT	0.148	0.190	0.429
CCTT	0.148	0.190	0.429
TCCT	0.479	0.627	**0.017**
TCTT	0.148	0.190	0.429
CTTC	0.155	0.211	0.283
CTCC	0.437	0.563	**0.043**
TCTC	0.176	0.225	0.374
CCCT	0.415	0.507	0.153
rs2284217—rs2284220			
GA	0.852	0.782	0.167
AG	0.690	0.704	0.897
AA	0.542	0.493	0.476
GG	0.563	0.486	0.235

*P* value < 0.05 were indicated in bold.

**Fig 4:**
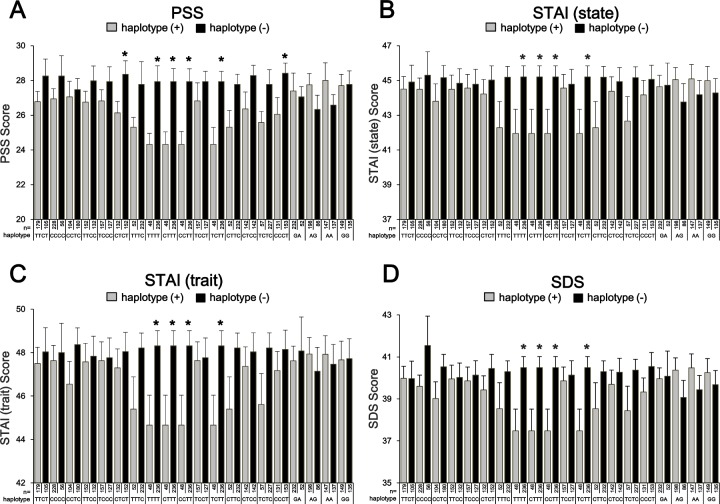
Psychological scale scores and *CRHR2* haplotypes. The psychological scales PSS (A), STAI (state) (B), STAI (trait) (C) and SDS (D) were shown. One-way ANOVA showed that the subjects with four haplotypes (T-T-T-T, C-T-T-T, C-C-T-T, and T-C-T-T) had significantly lower score than the others in all psychological scales. The four haplotype carriers were all the same individuals. In addition, in PSS, two haplotypes (C-T-C-T and C-C-C-T) were similarly significant.

We tested whether the psychological scale scores were different by haplotypes using One-way ANOVA. The result was shown in **Fig 4**. The psychological scales PSS (A), STAI (state) (B), STAI (trait) (C) and SDS (D) were shown. The carriers with four haplotypes (T-T-T-T, C-T-T-T, C-C-T-T, and T-C-T-T) had significantly lower scores than the others in all psychological scales. The four haplotype carriers were all the same individuals. In addition, in PSS, two haplotypes (C-T-C-T and C-C-C-T) were similarly significant.

We tested whether the psychological scale scores were different by haplotypes between the IBS patients and controls using two-way ANOVA. The result was shown in **Table 5.** In PSS, group effect (IBS vs. controls) was significant in six haplotypes. However, there was no group effect in other psychological scales. Haplotype effect was significant in all psychological scales. In STAI (trait), there was a significant group × haplotype interaction (*P* = 0.021). The four haplotype carriers (T-T-T-T, C-T-T-T, C-C-T-T, and T-C-T-T) were all the same individuals.

**Table 5:** Psychological state and *CRHR2* SNPs in IBS patients and controls.

Haplotype	IBS vs. controls (*P* value)	haplotype effect (*P* value)		group-haplotype interaction (*P* value)
PSS				
rs4722999—rs2267710				
TTTC	0.047	0.035		ns
TTTT*	0.041	0.005		ns
CTTT*	0.041	0.005		ns
CCTT*	0.041	0.005		ns
TCTT*	0.041	0.005		ns
CTTC	0.047	0.035		ns
CTCC	ns	0.041		ns
CCCT	ns	0.014		ns
STAI (state)				
rs4722999—rs2267710				
TTTC	ns	0.035		ns
TTTT*	ns	0.020		ns
CTTT*	ns	0.020		ns
CCTT*	ns	0.020		ns
TCTT*	ns	0.020		ns
CTTC	ns	0.035		ns
STAI (trait)				
rs4722999—rs2267710				
TTTT*	ns	0.020	ns	
CTTT*	ns	0.020	ns	
CCTT*	ns	0.020	ns	
TCTT*	ns	0.020	ns	
rs2284217—rs2284220				
GG	ns	ns	0.021	
SDS				
rs4722999—rs2267710				
TTTT*	ns	0.012	ns	
CTTT*	ns	0.012	ns	
CCTT*	ns	0.012	ns	
TCTT*	ns	0.012	ns	

Data without significance were not shown.

*: The four haplotype carriers (T-T-T-T, C-T-T-T, C-C-T-T, and T-C-T-T) were all the same individuals.

## Discussion

The present study is the first to show an association between IBS and SNPs/haplotypes of the *CRHR2* gene in the Japanese population. These data suggest that *CRHR2* gene polymorphisms and haplotypes are related to the pathophysiology of IBS.

Loss of CRHR2 leads to increased inflammation, delayed healing, and exacerbates the inflammatory insult [47–49]. Akiba et al. reported that peripheral CRHR2 activation induces colonic hyperemia through nitric oxide synthesis without involving prostaglandin synthesis or sensory nerve activation and may protect the colonic mucosa [50]. In a recent study, low grade inflammation was observed in the colonic mucosa of IBS patients [51]. We found that *rs4722999* and *rs3779250* were positively associated with IBS in terms of genotype frequency. Especially, for both SNPs, CT carriers were observed significantly more often in IBS patients. Ishitobi et al. investigated the association of six SNPs of *CRHR2* with MDD and panic disorder [36]. They reported that *rs3779250* was associated with MDD, but *rs4722999* and the other SNPs were not [36]. In this study, major allele carriers were observed frequently in MDD patients. Similarly, in our study, the frequency of the major allele of *rs3779250* was significantly different between the patients and controls. Like IBS, MDD is worsened by psychosocial stress with dysfunction of the HPA axis. From these results, the major allele of *rs3779250* may be a risk allele for stress-related diseases. However, the C allele of *rs3779250* is observed in approximately 77% of the normal population. Therefore, the increased frequency of the major allele of *rs3779250* alone cannot explain the relationship between *CRHR2* SNPs and IBS. Further study of other factors (e.g., other genetic polymorphisms) of IBS is required.

In this study, there was no significant difference in perceived stress, anxiety, and depression between the IBS patients and controls. Our previous report [52] and the results of others [53] showed that these factors are higher in IBS patients than in controls. By contrast, IBS patients were found to be neither depressive nor anxious in another study from our laboratory [54]. Therefore, IBS patients in this study were psychologically close to normal, but were physically stressed. This notion is supported by the fact that statistical analysis of stress, anxiety, and depression including *CRHR2* genotypes with group (IBS patients vs. controls) showed significant differences. The relatively complex association between genotypes and IBS suggests a modifying role for CRHR2 in the pathophysiology of stress.

In seven SNP which we selected, six SNPs are in the intronic region. Only rs2240403 is in the exon region, but it is the synonymous SNP which does not cause amino acid substitution. In this study, there were significant differences in the genotype distribution of both groups in rs472299 and rs377950. In addition, there were significant interaction in association between genotype and group (IBS patients vs. controls) in psychological condition in rs2190242 and rs2284220. These SNPs are in intronic region, and the domain is spliced during transcription to mRNA. Therefore those SNPs are less likely to have an influence on the receptor expression directly. However, the SNPs influencing modulation of the psychological condition that is in vicinity of these SNP in the linkage disequilibrium in IBS may exist.

We found a haplotype of four SNPs (*rs4722999*, *rs3779250*, *rs2240403*, and *rs2267710*) and two SNPs (*rs2284217* and *rs2284220*) in strong LD (D′ > 0.90). In this study, SNPs and haplotypes of the *CRHR2* gene were significantly different between IBS patients and controls. Sato et al. reported that genetic polymorphisms and T-A-T haplotypes of *CRHR1* mediate IBS and related bowel patterns [37]. Guillaume et al. reported that childhood sexual abuse and childhood emotional neglect interacted with *CRHR1* and *CRHR2* gene polymorphisms, respectively, to modulate adult decision-making in a cohort of suicide attempters [55]. Mahajan et al. reported that the balanced and coordinated expression of CRHR1 and CRHR2 is required for the proper regulation of urocortins in a rodent model of Crohn's colitis [56]. Therefore, haplotypes of both *CRHR1* and *CRHR2* may be related to the features of IBS.

We found that the psychological state varied from *CRHR2* genotypes and haplotypes in IBS patients and controls. Especially, we discovered that the group with one of four haplotypes (T-T-T-T, C-T-T-T, C-C-T-T, and T-C-T-T) in *CRHR2* showed a significantly lower score in perceived stress, anxiety, and depression in comparison with the group that did not have one of these haplotypes. In these four haplotypes, the common part is the T-T haplotype that consists of the T allele of rs2240403 and T allele of rs2267710. From this finding, it is suggested that having both these T alleles acts protectively against perceived stress and anxiety. To the best of our knowledge, this analysis of these four haplotypes of the *CRHR2* gene has not been performed before [35, 36]. This is a novel finding and further studies investigating the association between the T-T haplotype and other stress-related disorders are warranted.

This study has several limitations. First, the sample size was relatively small. Particularly, there were small numbers of subjects with the minor allele of *rs2240403* and the data had a wide standard error. However, our study has the strength of a higher precision of genome analyses than a genome-wide association study. Moreover, recently published genomic studies [57, 58] contained smaller number of participants than our study. Replication with a larger sample is necessary. Second, all subjects in this study were Japanese. Villafuerte et al. reported that the *CRHR2* gene was unlikely to be involved in the genetic liability underlying HPA axis dysfunction and mood disorders [59]. Therefore, there may be ethnic differences in the function of *CRHR2*. Third, the molecular biologic differences in the *CRHR2* gene polymorphisms examined in this study are not known. It is necessary to investigate any changes of function *in vivo* and behavior associated with the different *CRHR2* gene polymorphisms in the future. Despite some limitations, the present study is the first to clarify the possible importance of *CRHR2* in the pathophysiology of IBS.

In conclusion, our findings support the hypothesis that genetic polymorphisms and haplotypes of *CRHR2* are associated with IBS. Further studies of IBS and the CRH system are warranted.
